# Metabolic response strategies of spring wheat under osmotic stress

**DOI:** 10.3389/fpls.2026.1766233

**Published:** 2026-02-10

**Authors:** Houxing Yan, Chunxiang Wang, Yaping Liu, Zihan Xu, Fei Lin, Mingxuan Liu, Chunwu Yang

**Affiliations:** Key Laboratory of Molecular Epigenetics of Ministry of Education, Northeast Normal University, Changchun, China

**Keywords:** drought stress, energy metabolism, metabolome, ROS scavenging, spring wheat

## Abstract

Spring wheat is predominantly cultivated in arid and semi-arid regions, where drought stress severely limits its productivity. However, the physiological and metabolic mechanisms underlying drought tolerance in spring wheat remain unclear. In this study, to explore drought tolerance mechanisms of spring wheat, we compared physiological and metabolomic responses of a drought-tolerant pure line BM14 and a widely grown variety NC4 to osmotic stress induced by PEG-6000. The results showed that BM14 exhibited stronger osmotic stress tolerance than NC4, maintaining higher relative water content, lower water loss, and more stable photosynthesis under osmotic stress. Under osmotic stress, BM14 displayed higher levels or activities of energy-related metabolites (carbohydrates), K^+^, SOD, and CAT. Particularly, under osmotic stress, leaf K^+^ content of BM14 was much higher than that of NC4, suggesting that enhanced K^+^ retention contributes to osmotic adjustment. Regarding non-enzymatic antioxidants, concentrations of many phenolic acids were increased by osmotic stress in the roots of BM14 but not in those of NC4, while the concentrations of flavonoids, phenolic acids, and B vitamins were significantly increased in BM14 leaves but not in NC4. Collectively, these findings indicated that regulation of K^+^ homeostasis, energy metabolism, and organ-specific ROS scavenging is closely associated with osmotic stress tolerance in BM14. The identified physiological traits and metabolic signatures may provide potential indicators and candidate targets for the selection and improvement of drought-tolerant spring wheat genotypes.

## 1 Introduction

Drought, one of the most complex and destructive natural disasters globally, is the predominant abiotic stress affecting agricultural production (Gong et al., 2020; Rezaei et al., 2023; Sato et al., 2024). Global warming may lead to more frequent and widespread drought, which will severely impair crop growth, development, and yield, thus posing a significant threat to global food security (Hou et al., 2024; Wang et al., 2024a). Consequently, the identification and utilization of drought-tolerant crop genotypes represent essential strategies for mitigating the adverse impacts of extreme climatic conditions on agricultural productivity. Spring wheat (*Triticum aestivum* L.) is predominantly cultivated in arid and semi-arid regions, where it is frequently exposed to water deficit conditions (Zhao, 2010). Screening and breeding of high-quality and drought-tolerant spring wheat cultivars are particularly important for food security. However, most of the drought research currently focuses on winter wheat, and little is known about mechanisms by which spring wheat resists drought stress.

The primary consequence of drought stress is water deficiency, which lowers water potential around the root system, leading to stomatal closure and reactive oxygen species (ROS) accumulation (Chaves et al., 2003; Fahad et al., 2017; Hasanuzzaman et al., 2012; Kim et al., 2025; Xiong and Zhu, 2002). Elevated ROS levels cause oxidative damage to nucleic acids, proteins, and lipids, ultimately inhibiting CO_2_ assimilation, plant growth, and yield formation (Ozturk et al., 2021; Rane et al., 2021). Plants respond to water deficiency through a range of complex metabolic regulatory mechanisms (Gupta et al., 2020; Haghpanah et al., 2024; Kaur and Asthir, 2017; Muhammad Aslam et al., 2022). Several hub metabolites play critical roles in crop drought tolerance. Free carbohydrates, soluble proteins, and free amino acids function as osmotic regulators, often in coordination with inorganic ions, to maintain cellular integrity (Ahmad and Majeti, 2012; Koyro et al., 2012; Raza et al., 2025; Traversari et al., 2018). Certain carbohydrates and free fatty acids, as substrates, participate in essential energy metabolism pathways that support osmotic regulation and biosynthesis of ROSs scavenger, thereby contributing to drought responses (Raza et al., 2025; Selinski et al., 2024; Zhang et al., 2024). Furthermore, phenolic acids, flavonoids, vitamins, glutathione, and unsaturated fatty acids act as non-enzymatic antioxidants, scavenging ROS and protecting cells from oxidative damage (Asensi-Fabado and Munne-Bosch, 2010; Farooq et al., 2013; Kosar et al., 2021; Raza et al., 2025, 2024).

Previous metabolomic studies have highlighted the importance of specific metabolites in drought tolerance. Guo et al. (2024) found that plant hormones, tryptamine derivatives, phenolamides, polyphenols, and flavonoids were positively related to rice (*Oryza sativa* L.) drought tolerance. Li et al. (2024) explored the metabolic responses of maize (*Zea mays* L.) seedlings to drought stress and found that proline, tryptophan, and phenylalanine were identified as playing key roles in the drought response. Chaturvedi et al. (2024) found that organic acids, amino acids, sugar alcohols, and succinic acid, citric acid, and pyruvate were highly correlated with drought tolerance of chickpea (*Cicer arietinum* L.). In annual ryegrass (*Lolium multiflorum* L.), Pan et al. (2018) identified the core metabolic pathways involved in drought tolerance, including lipids, amino acids, organic acids, amines, and pyridines and their derivatives. A wheat variety Norin61 exhibited significant changes in amino acids, nucleosides, and organic acids during the flowering stage under drought stress, with His, Val, Trp, and Ile identified as drought biomarkers (Itam et al., 2020). Guo et al. (2020) found that, under osmotic stress, compared with the drought-sensitive winter wheat variety, the drought-tolerant variety showed higher levels of phenolics, amino acids, alkaloids, organic acids, and flavonoids in leaves. Wonneberger et al. (2025) found that amino acids and polyamines may play important roles in drought tolerance of a Nordic spring wheat variety, and an Iran spring wheat variety showed enhanced levels of major purine bases (adenine and guanine), proline, tryptophan, and branched chain amino acids under drought conditions (Michaletti et al., 2018). Compared with winter wheat, spring wheat differs in growth habit, phenological development, and stress exposure during critical growth stages, yet most drought-related studies have primarily focused on winter wheat. As a result, the physiological and metabolic responses underlying drought tolerance in spring wheat remain relatively underexplored. In this study, polyethylene glycol (PEG)-6000 was used to mimic osmotic stress (physiological drought conditions). A drought-tolerant spring wheat pure line BM14 was selected as the experimental material. By integrating physiological measurements with widely targeted metabolomic profiling, this study aimed to characterize genotype-specific metabolic and physiological responses to osmotic stress and to identify metabolic features associated with enhanced stress tolerance in spring wheat.

## 2 Materials and methods

### 2.1 Plant material and treatment

We selected the drought-tolerant spring wheat pure line BM14 (Bamai14) as the experimental material, and a widely cultivated spring wheat variety NC4 (NingChun4) as the control. Seeds of both genotypes were kindly provided by Dr. Lei Yang (Bayannur Academy of Agricultural Sciences, Bayannur, China). After surface sterilization and germination in Petri dishes, uniformly germinated seeds with coleoptile approximately 1 cm long were transferred to hydroponic boxes containing 1/2×Hoagland solution. The seedlings were grown in a controlled growth chamber (24 °C/17 °C, 16 h light/8 h dark), with the nutrient solution replaced daily. After four days of cultivation, the seedlings were treated with 1/2×Hoagland solution supplemented with 15% (w/v) PEG-6000 for an additional 10 days, and control plants were cultivated with 1/2×Hoagland solution without PEG. PEG-6000 was widely applied to mimic osmotic stress (Chen et al., 2025; Wang et al., 2025; Yan et al., 2025), and a concentration of 15% was used because preliminary experiments revealed markedly different responses of the two varieties to PEG-6000 of this concentration without causing irreversible damage. After 10 days of treatments, plant phenotypic traits were documented via photographing, and then various physiological and biochemical parameters were measured. The second fully expanded leaf from each biological replicate was collected and stored at −80 °C for metabolomic analysis. All experiments were conducted with three biological replicates, each replicate consisting of a pooled sample from 8–12 plants from a box.

### 2.2 Measurement of physiological indicators

Relative water content (RWC) was determined following the method of Li et al. (2023), with minor modifications. Fresh weight (FW), saturated weight (SW, after immersion in distilled water), and dry weight (DW, after oven-drying) were recorded. RWC (%) = (FW - DW)/(SW - DW) × 100%. The rate of water loss (RWL) was measured according to Jiang et al. (2021). Fresh weight (FW_1_), weight after air-drying at room temperature in the dark for 5 h (FW_2_), and dry weight (DW) were recorded. RWL (g·g^-^¹·h^-^¹) = (FW_1_ - FW_2_)/(DW × t). Gas exchange and chlorophyll fluorescence parameters were measured on fully expanded mature leaves using a portable photosynthesis and fluorescence system (LI-6800; LI-COR Biosciences, Lincoln, NE, USA). Measurement conditions were maintained at a light intensity of 1200 μmol m^−2^·s^−1^, a CO_2_ concentration of 400 μmol·mol^−1^, and a relative air humidity of 55%. Chloroplast pigment contents were determined following the method of Ni et al. (2009). Dry samples were digested with HNO_3_ at 120°C for measurements of mineral elements. Potassium (K^+^) content was determined using an atomic absorption spectrophotometer (TAS-990; Beijing Puxi Instrument Co., China). The contents of calcium (Ca), and magnesium (Mg), iron (Fe), manganese (Mn) mineral elements were analyzed using inductively coupled plasma atomic emission spectrometry (ICP-AES). Electrolyte leakage rate was assessed using a conductivity meter (DDS-307A; LeiMagnet, China). Superoxide anion (O_2_^-^) content was measured according to the method of Wan et al. (2014). The activities of superoxide dismutase (SOD), peroxidase (POD) and catalase (CAT) were determined following the methods of Zhou et al. (2021) and Chen and Zhang (2016). One unit (U) of enzyme activity was defined as a change in absorbance of 0.01 per minute under the assay conditions, and enzyme activities were expressed as U·g^-^¹ fresh weight (FW). Malondialdehyde (MDA) content was measured according to Tang (1999) and Fu et al. (2022). Soluble protein content was determined using the Coomassie Brilliant Blue method (Bradford, 1976). The activities of sucrose phosphate synthase (SPS) and sucrose synthase (SuSy) were determined spectrophotometrically (Verma et al., 2011) and expressed as nmol·g^-^¹·min^-^¹ FW.

### 2.3 Widely targeted metabolomics analysis

#### 2.3.1 Sample preparation and extraction

A widely targeted metabolomics analysis was performed with the methods of Chen et al. (2013) and Gu et al. (2024). The samples were vacuum freeze-dried and then a sample of 50 mg was extracted with 1,200 μL of pre-cooled 70% methanol. After vortexing, samples were centrifuged at 12,000 rpm for 3 min, and the supernatants were transferred to injection vials for subsequent UPLC–MS/MS analysis.

#### 2.3.2 Metabolite acquisition conditions

The metabolites were analyzed with a UPLC-ESI-MS/MS system (QTRAP, AB SCIEX). Chromatographic separation was performed on a UPLC system equipped with an Agilent SB-C18 column (1.8 μm, 2.1 × 100 mm). The column temperature was maintained at 40°C, and the injection volume was 2 μL. The ESI source parameters were set as follows: source temperature, 550°C; ion spray voltage, 5,500 V (positive mode) and –4,500 V (negative mode). Data were acquired in multiple reaction monitoring (MRM) mode using nitrogen as the collision gas. Declustering potential and collision energy were optimized for each transition, and a scheduled MRM strategy was applied.

#### 2.3.3 Metabolite identification and quantification

Metabolites were identified by matching MS/MS spectra, retention time (RT), and ion transition parameters (Q1, Q3, DP, and CE) against a curated in-house database (MWDB, MetWare Database) established by MetWare Biological Science and Technology Ltd, China (https://www.metware.cn). According to MWDB criteria, metabolites were classified into three confidence levels: level 1, MS/MS spectral and RT matching with similarity ≥0.7; level 2, MS/MS and RT matching with similarity between 0.5 and 0.7; and level 3, matching of Q1/Q3, RT, and optimized MS parameters. Metabolite quantification was performed in MRM mode using MultiQuant software (SCIEX), and peak areas were reported as relative abundances. Internal standards were used to monitor extraction efficiency and instrument stability.

#### 2.3.4 Quality control and data processing for metabolomics data

Quality control (QC) samples were prepared by pooling equal aliquots of all extracts and injected every ten runs to monitor analytical stability. Retention time and signal intensity stability were evaluated using total ion current (TIC) overlays of QC samples. Raw peak areas were normalized to sample dry weight and internal standards. Missing values were imputed using one-fifth of the minimum detected value for each metabolite. Metabolites with coefficients of variation (CV) < 0.5 in QC samples were retained for analysis. Standard workflows implemented in MetaboAnalyst 4.0 were applied for orthogonal partial least squares-discriminant analysis (OPLS-DA), and variable importance in projection (VIP) values were derived from OPLS-DA models.

### 2.4 Statistical analyses

Data are presented as means ± standard deviation (SD). Graphical visualizations were performed using Origin 2025 (OriginLab Corporation, Northampton, MA, USA). Physiological traits were analyzed using one-way analysis of variance (ANOVA) under a completely randomized design. Least significant difference (LSD) test (*P* < 0.05) was used for mean comparisons with SPSS software (IBM SPSS Statistics, Armonk, NY, USA). Principal component analysis (PCA) for metabolic data was performed with Origin 2025. Differentially accumulated metabolites (DAMs) were initially screened using the criteria VIP > 1, |log2 (fold change) | > 1, and *P* < 0.05 (Student’s *t*-test). Stress-upregulated metabolites unique to BM14 (SUMs) were further identified based on Benjamini-Hochberg false discovery rate (FDR)-adjusted *P* < 0.05, VIP > 1, and |log2 (fold change) | > 1.

## 3 Results

### 3.1 Physiological indicators

**Figure 1:**
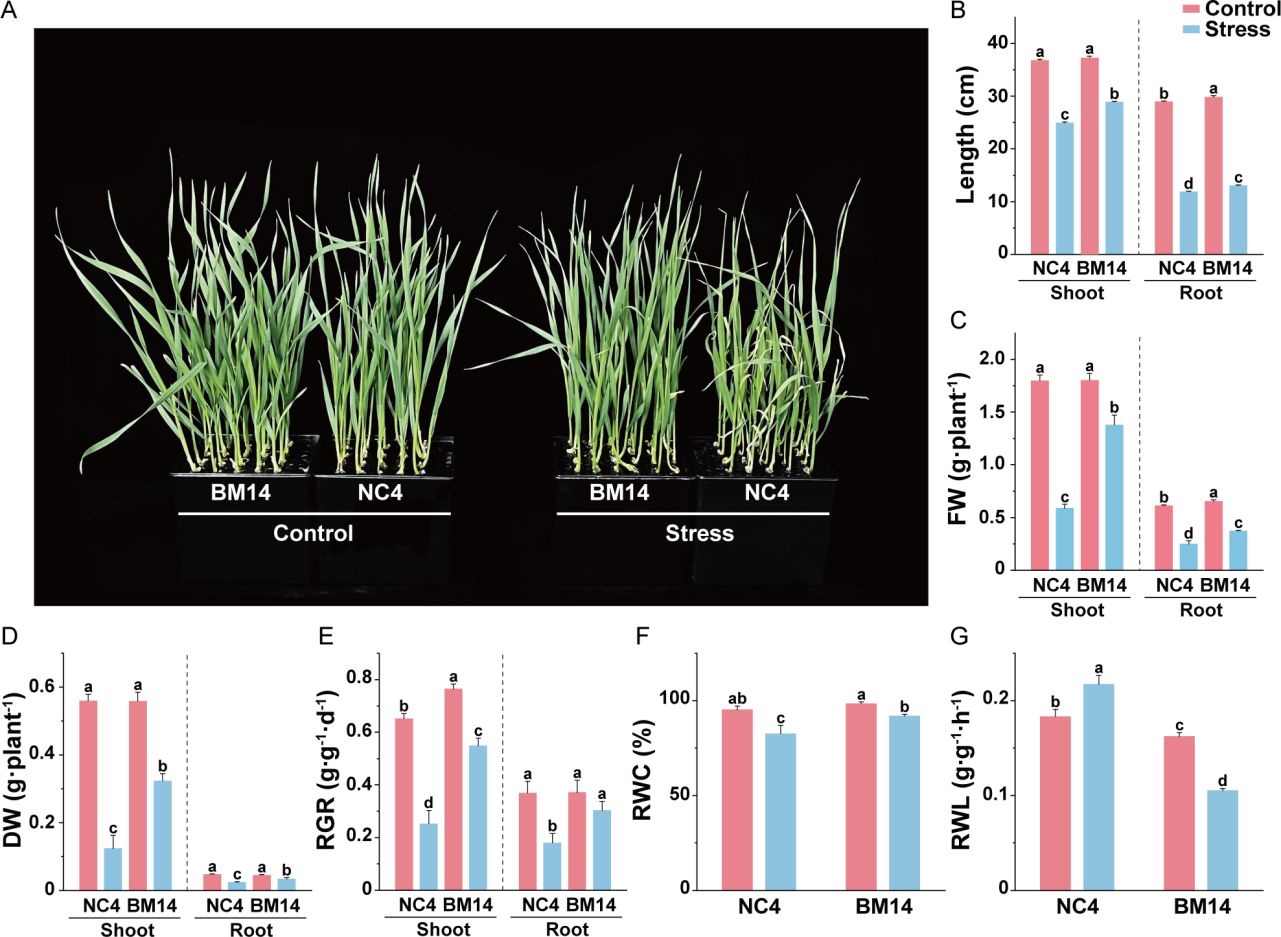
Effects of osmotic stress on growth phenotype and physiological parameters of spring wheat. **(A)** Phenotypes of BM14 and NC4 under control and stress treatments; **(B)** Plant height and root length; **(C)** FW, fresh weight; **(D)** DW, dry weight; **(E)** RGR, relative growth rate; **(F)** RWC, relative water content; **(G)** RWL, rate of water loss. Data are presented as means ± SD of three biological replicates (n = 3). Different letters indicate significant differences among treatments at the same organ (LSD test, P < 0.05). FW, fresh weight; DW, dry weight; RGR, relative growth rate; RWC, relative water content; RWL, rate of water loss.

**Figure 2:**
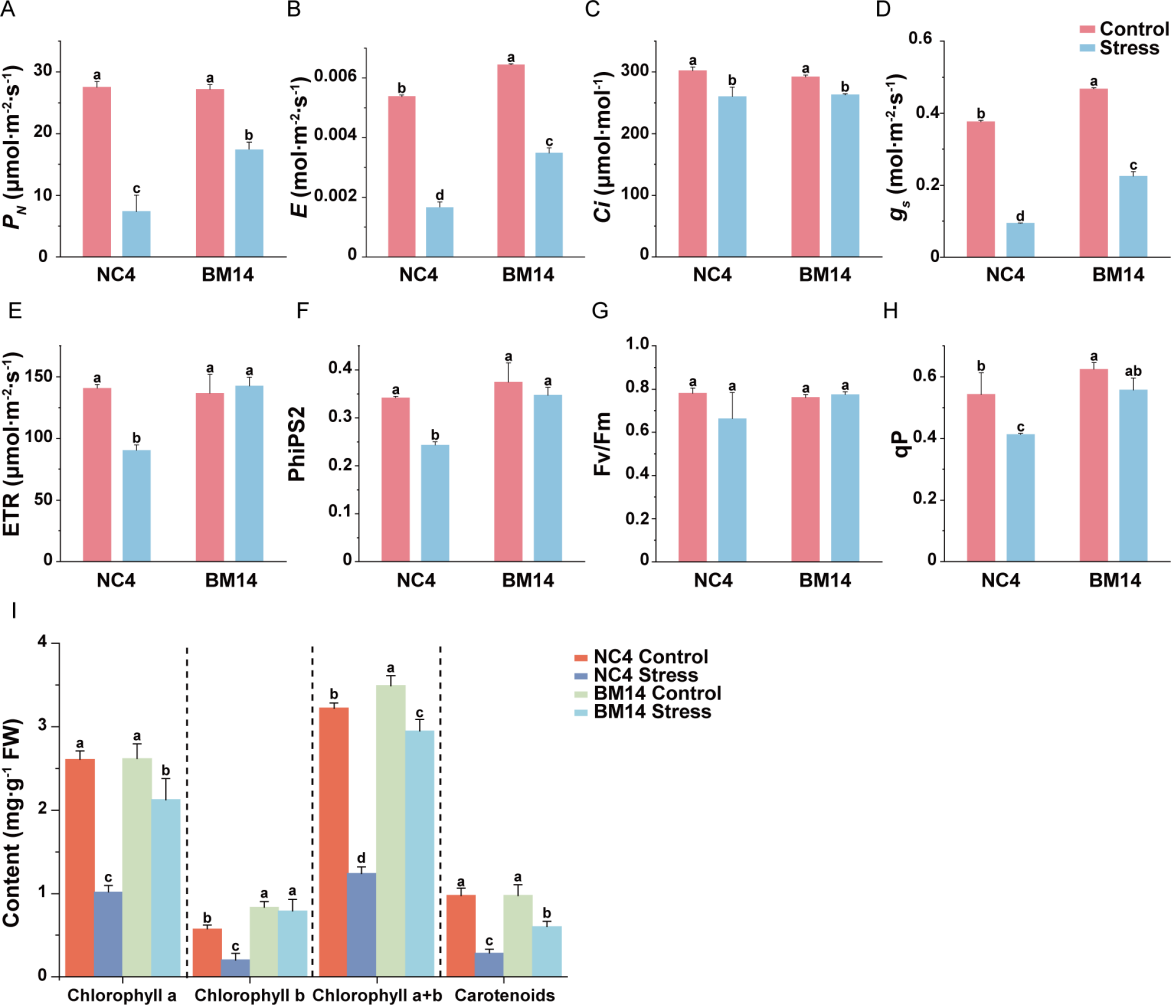
Effect of osmotic stress on gas exchange, chlorophyll fluorescence parameters and pigment contents in spring wheat. **(A)***P*_N_, net photosynthetic rate; **(B)***E*, transpiration rate; **(C)***Ci* intercellular CO2 concentration; **(D)***gs*, stomatal conductance; **(E)** ETR, electron transfer efficiency; **(F)** PhiPS2, real quantum efficiency of PSII; **(G)** Fv/Fm, maximum photochemical quantum efficiency of PSII; **(H)** qP, photochemical quenching coefficient; **(I)** Pigment contents. Data are presented as means ± SD of three biological replicates (n = 3). Different letters indicate significant differences among treatments (LSD test, P<0.05).

**Figure 3:**
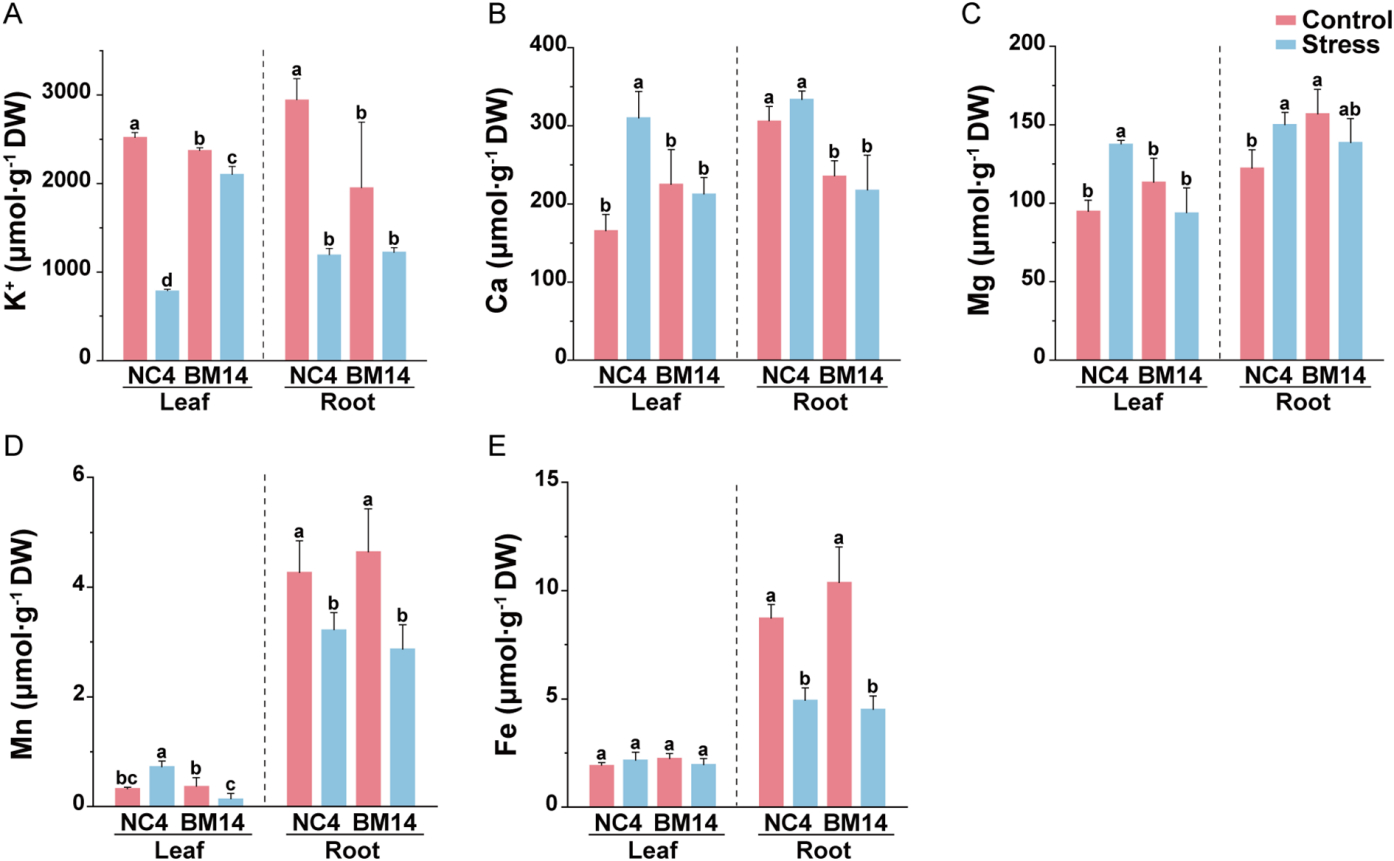
Effects of osmotic stress on nutrient elements of spring wheat. **(A)** K^+^; **(B)** Ca; **(C)** Mg; **(D)** Mn; **(E)** Fe. Data are presented as means ± SD of three biological replicates (n = 3). Different letters indicate significant differences among treatments at the same organ (LSD test, P<0.05).

**Figure 4:**
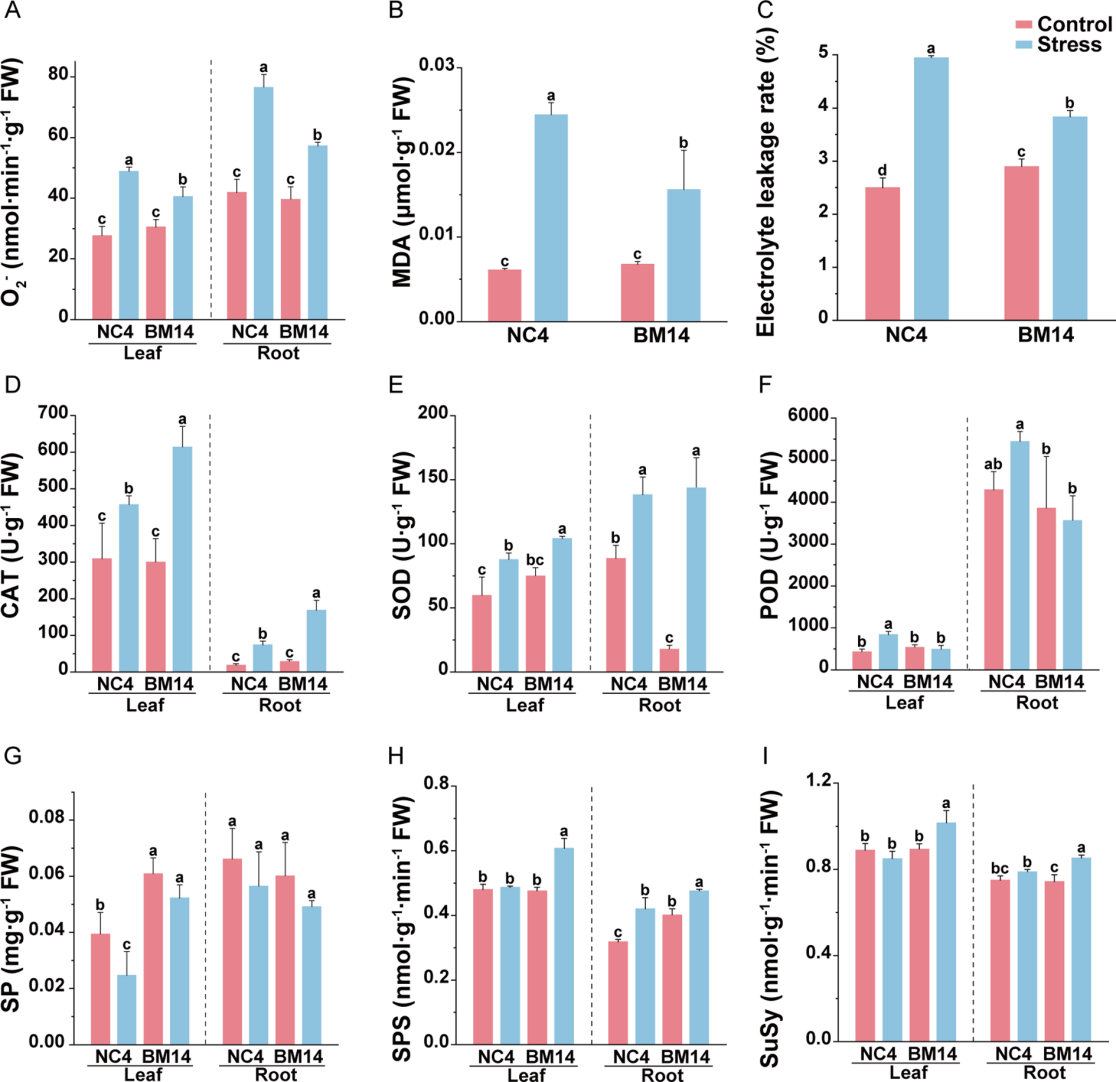
Effects of osmotic stress on enzyme activities involved in sucrose metabolism and antioxidant system of spring wheat. **(A)** O_2_^-^content; **(B)** MDA content; **(C)** Electrolyte leakage rate; **(D)** CAT activity; **(E)** SOD activity; **(F)** POD activity; **(G)** Soluble protein content; **(H)** SPS activity; **(I)** SuSy activity. Data are presented as means ± SD of three biological replicates (n = 3). Different letters indicate significant differences among treatments at the same organ (LSD test, P<0.05).

After 10 days of osmotic stress treatment, no significant differences were observed between the drought-tolerant spring wheat pure line BM14 and the widely cultivated spring wheat variety NC4 under control conditions. However, under osmotic stress, BM14 exhibited better growth performance compared to NC4 (**Figure 1A**). Osmotic stress significantly lowered plant height and biomass, as well as the relative growth rates in both varieties, with less reduction in BM14 than in NC4 (**Figures 1B-E**). After the stress treatment, BM14 exhibited a smaller reduction in RWC than NC4 (**Figure 1F**). Under osmotic stress, RWL was much lower in BM14 than in NC4 (**Figure 1G**). BM14 exhibited a stronger leaf water retention capacity and a lower water loss rate, which contributed to maintaining a more stable intracellular water status. Osmotic stress displayed much stronger inhibitory effects on photosynthesis in NC4 than in BM14 (**Figure 2**). Chlorophyll fluorescence parameters of BM14 were unaffected by osmotic stress, and its photosynthetic pigment content showed a slight decline under the stress condition. In contrast, NC4 exhibited a significant reduction in both chlorophyll fluorescence parameters and pigment content. Ion homeostasis was also disrupted under osmotic stress, leaf K^+^ content in NC4 decreased to 31% of the control, while in BM14 it decreased to 89% of the control (**Figure 3A**). Although the K^+^ content in the roots of both varieties decreased under stress condition, there was no significant difference between the two varieties under osmotic stress. Other mineral elements in BM14 exhibited slight changes after the stress treatment, whereas those mineral elements in NC4 displayed more pronounced upregulation or downregulation (**Figures 3B-E**). Osmotic stress elevated O_2_^-^ levels in both genotypes, with lower enhancement in BM14 than in NC4 (**Figure 4A**). Consistently, changes in MDA content (**Figure 4B**) and electrolyte leakage rate (**Figure 4C**) indicated that BM14 experienced less oxidative damage. Under osmotic stress, in the leaves, the CAT and SOD activity of BM14 was 0.34 times and 0.18 higher than that of NC4, respectively. In the roots, CAT activity in BM14 was 1.28 times higher than that in NC4 under osmotic stress (**Figures 4D–F**). Under osmotic stress, soluble protein content significantly decreased in the leaves of NC4, while remaining unchanged in BM14 (**Figure 4G**). Additionally, SPS and SuSy activity were significantly increased in the roots of BM14 after osmotic stress treatment, whereas no significant changes were detected in NC4 (**Figures 4H-I**).

### 3.2 Metabolic profiling under osmotic stress

**Figure 5:**
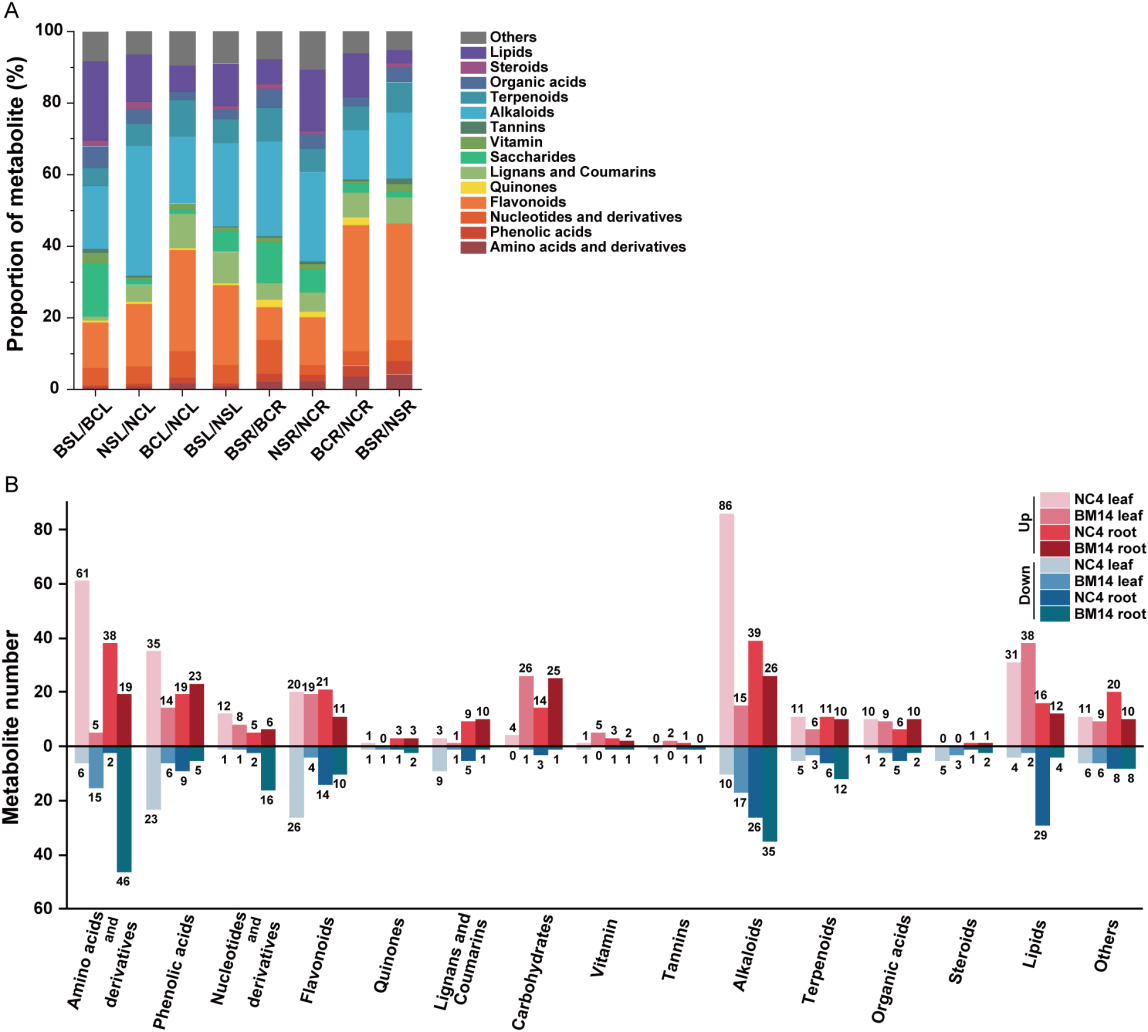
Comparison of two spring wheat genotypes in metabolic response. **(A)** The proportion of each metabolite type for different comparisons. BCL, BM14 leaves under control conditions; BSL, BM14 leaves under osmotic stress; NCL, NC4 leaves under control conditions; NSL, NC4 leaves under osmotic stress; BCR, BM14 roots under control conditions; BSR, BM14 roots under osmotic stress; NCR, NC4 roots under control conditions; NSR, NC4 roots under osmotic stress; **(B)** Number of upregulated and downregulated metabolites caused by osmotic stress for the two spring wheat genotypes. The abscissa represents the metabolite types, with red representing upregulated and blue representing downregulated.

A widely targeted metabolomics approach was employed to characterize metabolic responses to osmotic stress in the leaves and roots of BM14 and NC4. A total of 1,716 metabolites were collectively detected in the leaves and roots of BM14 and NC4. These metabolites encompassed a wide range of compound classes, including 221 alkaloids, 220 amino acids and derivatives, 373 flavonoids, 81 lignans and coumarins, 69 nucleotides and derivatives, 59 organic acids, 142 lipids, 193 phenolic acids, 19 quinones, 8 steroids, 9 tannins, 104 terpenoids, 58 carbohydrates, 17 vitamins, and 143 compounds classified as others. Based on metabolite identification confidence criteria in the MWDB database, the detected metabolites were classified into three confidence levels. Among them, 545 metabolites were assigned to level 1, 314 to level 2, and 857 to level 3 (**Supplementary Table 1**), indicating a high overall reliability of metabolite annotation. The QC plots were presented in **Supplementary Figure 1**. Principal Component Analysis (PCA) revealed distinct separations between treatment groups, indicating significant metabolite changes in response to the stress (**Supplementary Figure 2**). To assist group discrimination, OPLS-DA was performed for each tissue and genotype. For a representative comparison, the OPLS-DA model explained a substantial proportion of the variance, with R²X = 0.447 and R²Y = 0.991, and showed acceptable predictive performance, as indicated by a Q² value of 0.888 (**Supplementary Figure 3**). Differentially accumulated metabolites (DAMs) were identified for each comparison based on the criteria of VIP > 1, |log2 (fold change) | > 1, and *P* < 0.05. The number and proportion of DAMs in each comparison group were shown in **Table 1** and **Figure 5**, and the detailed information was listed in the **Supplementary Table 1**. In the comparison between BM14 and NC4 leaves under control conditions, 279 DAMs were identified, whereas osmotic stress induced 645 DAMs between BM14 and NC4 leaves. Analysis of DAM revealed distinct metabolic response patterns between the two genotypes. In both organs of BM14, osmotic stress induced a notable accumulation of carbohydrates and lipids, but it downregulated the levels of many alkaloids and amino acid or amino acid derivatives. In contrast, in both organs of NC4, osmotic stress upregulated the accumulation levels of many alkaloids and amino acid or amino acid derivatives, suggesting distinct metabolic response strategies between the two genotypes.

**Table 1:** Type and number of differentially accumulated metabolites.

Class metabolites	BSL/ BCL	NSL/ NCL	BCL/ NCL	BSL/ NSL	BSR/ BCR	NSR/ NCR	BCR/ NCR	BSR/ NSR
Amino acids and derivatives	20	67	92	112	65	40	32	31
Phenolic acids	20	58	16	60	28	28	21	32
Nucleotides and derivatives	9	13	13	25	22	7	8	11
Flavonoids	23	46	50	107	21	35	69	62
Quinones	1	2	1	3	5	4	4	0
Lignans and Coumarins	2	12	17	42	11	14	14	14
Carbohydrates	27	4	2	28	26	17	5	3
Vitamins	5	2	3	5	3	4	1	4
Tannins	2	1	0	1	1	2	1	3
Alkaloids	32	96	33	112	61	65	27	35
Terpenoids	9	16	18	32	22	17	13	16
Organic acids	11	11	4	13	12	11	5	8
Steroids	3	5	0	5	3	2	0	2
Lipids	40	35	13	57	16	45	24	7
Others	15	17	17	43	18	28	12	10
All	219	385	279	645	314	319	236	238
Number of upregulated	157	286	211	380	168	206	89	83
Number of downregulated	62	99	68	265	146	113	147	155

BCL, BM14 leaves under control conditions; BSL, BM14 leaves under osmotic stress; NCL, NC4 leaves under control conditions; NSL, NC4 leaves under osmotic stress; BCR, BM14 roots under control conditions; BSR, BM14 roots under osmotic stress; NCR, NC4 roots under control conditions; NSR, NC4 roots under osmotic stress.

### 3.3 Analysis of stress-upregulated metabolites

To elucidate the genotype-specific metabolic regulation mechanisms of the drought-tolerant spring wheat under osmotic stress, we focused on stress-upregulated metabolites unique to BM14 (SUM). According to the statistical test criterion FDR < 0.05, VIP > 1, and |log2 (fold change) | > 1, we identified SUMs if the levels of the metabolites were elevated in BM14 not in NC4 as well as the levels of the metabolites were upregulated in both genotypes but BM14 displayed higher levels than NC4 under the stress condition. Detailed information of SUMs was displayed in **Table 2**; **Supplementary Table 1**. Carbohydrates were the primary SUMs, with 14 ones in leaves and 15 ones in roots. Besides carbohydrates, a number of flavonoids and phenolic acids were identified as SUM in leaves, while many of phenolic acids, lignans and coumarins were identified as SUM in roots.

**Table 2:** The number of SUM for each metabolite type.

Stress-upregulated metabolites	Leaf	Root
Carbohydrates	14	15
Vitamin	3	1
Others	4	5
Lipids	3	6
Flavonoids	6	4
Phenolic acids	3	13
Organic acids	3	6
Terpenoids	1	3
Nucleotides and derivatives	1	4
Alkaloids	2	6
Tannins	1	0
Amino acids and derivatives	0	5
Lignans and Coumarins	1	8
Quinones	0	1
Steroids	0	0
total	42	77

### 3.4 Analysis of changes in major SUMs under stress

**Figure 6:**
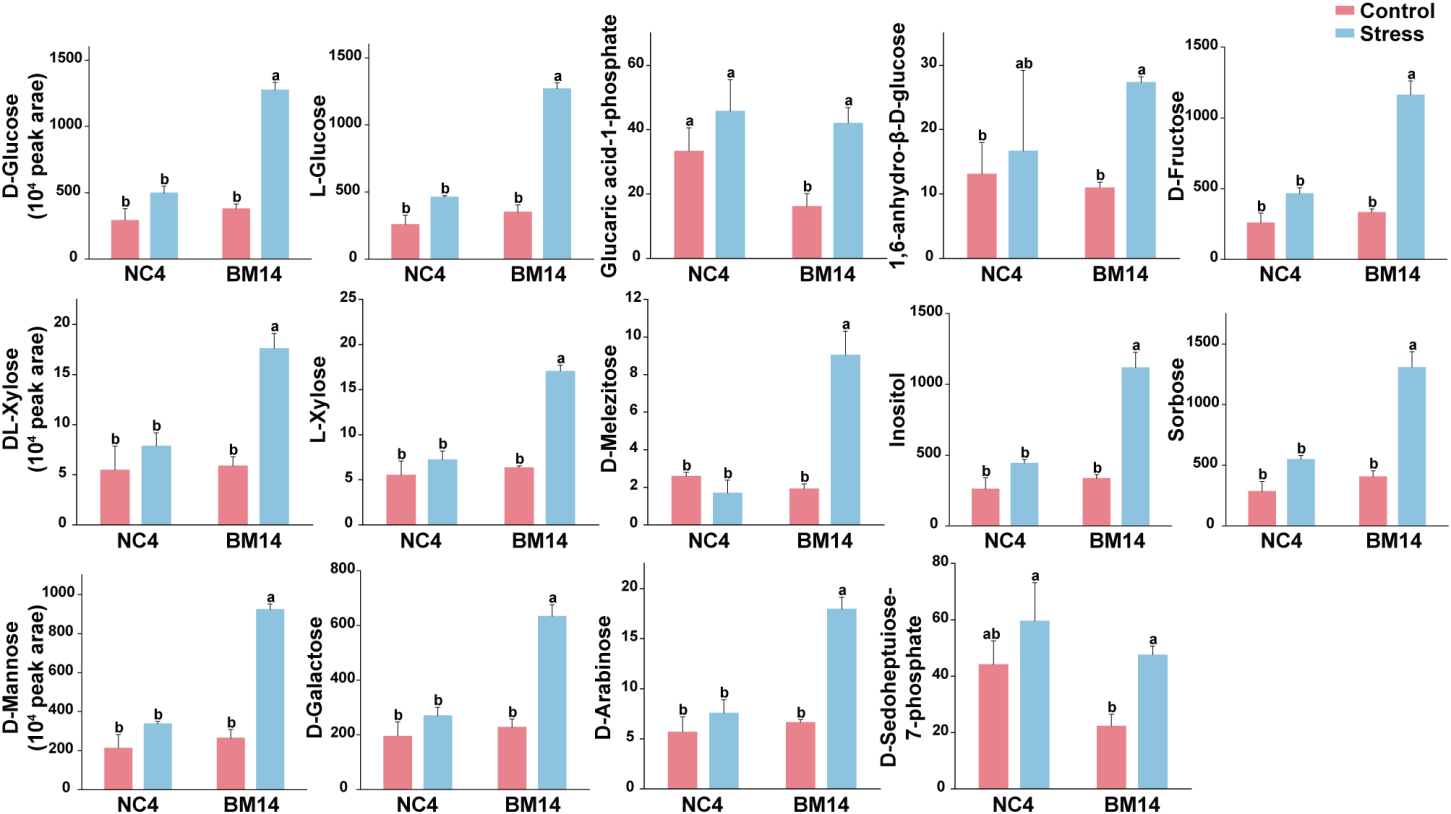
Effects of osmotic stress on relative concentrations of carbohydrates in leaves. Data are presented as means ± SD of three biological replicates (n = 3). Different letters indicate significant differences among treatments (VIP > 1, |log_2_(FC)| > 1, and FDR < 0.05).

**Figure 7:**
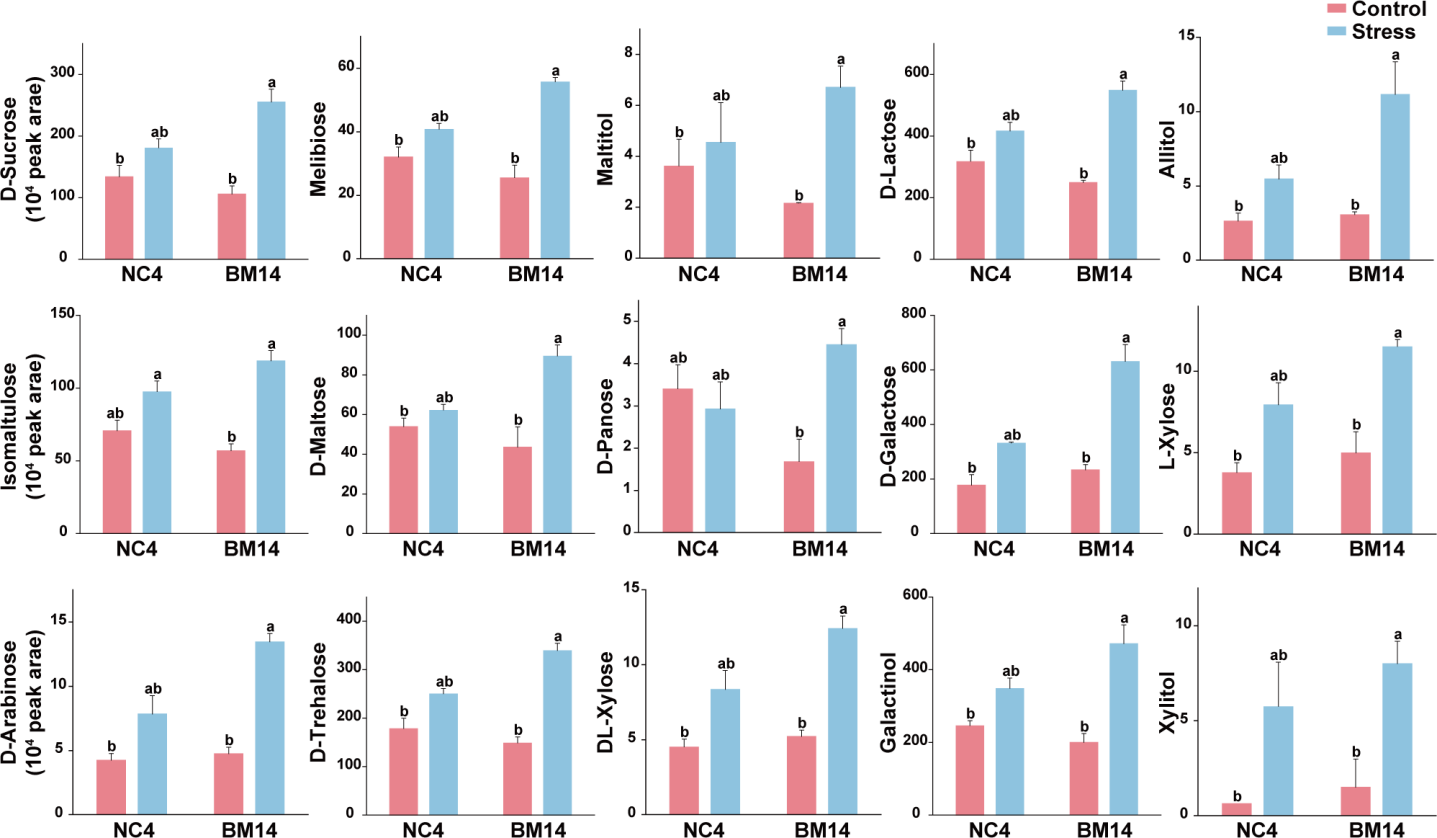
Effects of osmotic stress on relative concentrations of carbohydrates in roots. Data are presented as means ± SD of three biological replicates (n = 3). Different letters indicate significant differences among treatments (VIP > 1, |log_2_(FC)| > 1, and FDR < 0.05).

**Figure 8:**
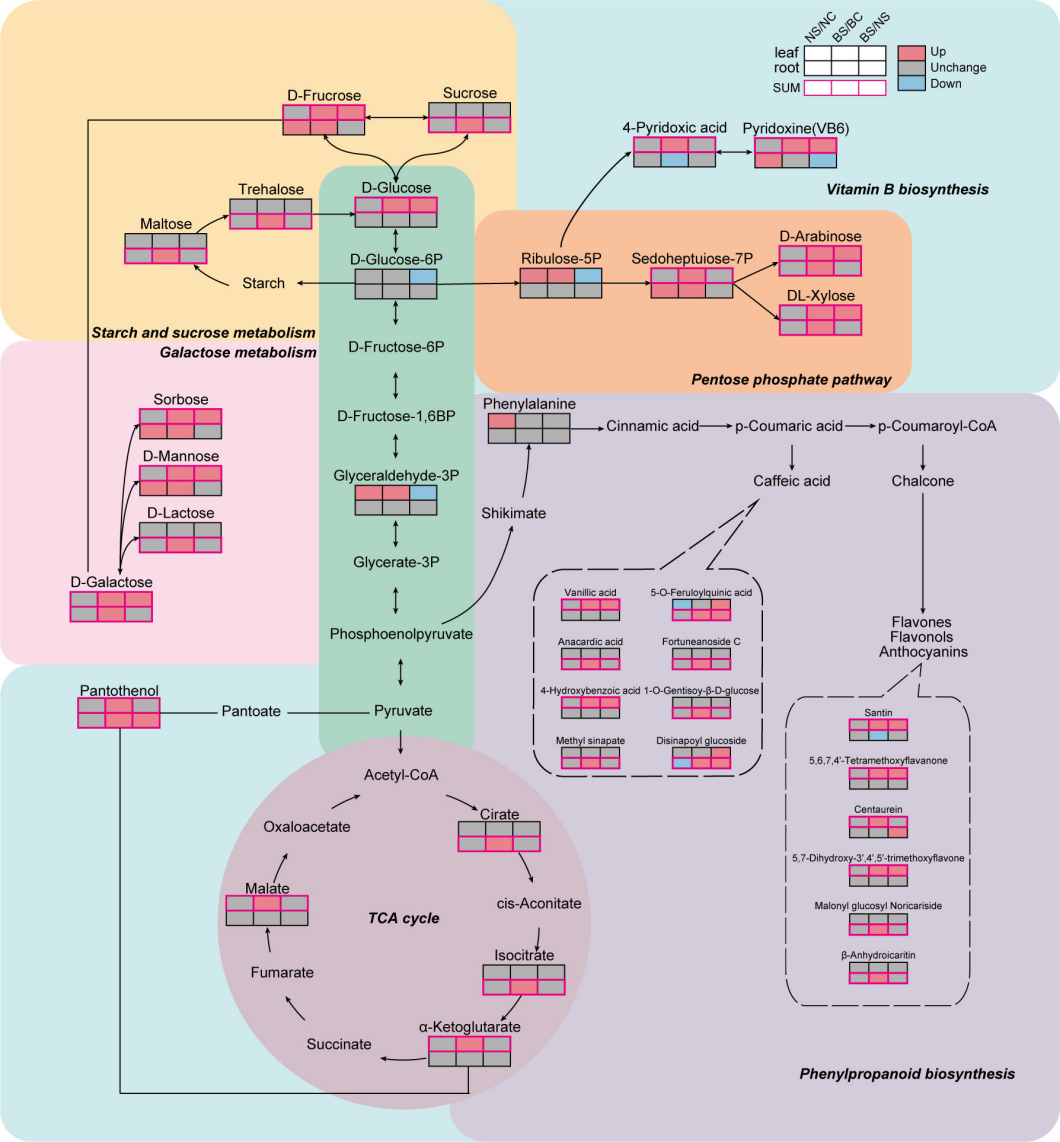
Comparison of metabolic response network of the two spring wheat genotypes under osmotic stress. The boxes below the metabolites indicate their changes caused by osmotic stress, the top three boxes represent leaf and the bottom three boxes represent root. Red, blue, and gray boxes represent upregulated, downregulated and unchanged, respectively. Metabolites highlighted with red borders indicate SUMs.

We discovered 14 and 15 carbohydrates in list of leaf and root SUM, respectively (**Figures 6**, **7**). The 14 carbohydrates in leaves mainly included D-arabinose, D-fructose, D-galactose, D-glucose, DL-xylose, D-mannose, Sorbose. Notably, 4 carbohydrates (D-arabinose, D-galactose, DL-xylose, L-xylose) were identified as SUMs in both leaves and roots, and all of them were upregulated in BM14 not in NC4. In the list of leaf SUMs, we discovered 3 organic acids, including citric acid glucoside, D-malic acid, and α-ketoglutaric acid (**Supplementary Figure 4**). In list of root SUM, we found 6 organic acids, including citric acid, citric acid diglucoside, citric acid glucoside, isocitric acid, malic acid-1-O-diglucoside and tartronate semialdehyde (**Supplementary Figure 4**). Among them, citric acid glucoside was identified as SUM in both organs. We discovered 6 flavonoids with SUM pattern in leaves, including 3 flavonols, 2 flavones, and 1 flavanone (**Supplementary Figure 5**). In SUM list of roots, we found 4 flavonoids, levels of which all were significantly upregulated in BM14 under osmotic stress but remained unchanged in NC4. Furthermore, 3 phenolic acids were upregulated in the leaves of BM14 but not in the leaves of NC4 (**Supplementary Figure 6**), and 13 phenolic acids were upregulated in the roots of BM14 but not in the roots of NC4. A few lipids were identified as SUM (**Supplementary Figure 7**). Additionally, levels of 3 B vitamins (pyridoxine, 4-pyridoxic acid, D-panthenol) were enhanced in the leaves of BM14 but not in the leaves of NC4 (**Supplementary Figure 8**). Only one B vitamin was identified as SUM in roots. To obtain an integrated view of metabolic adjustments associated with osmotic stress tolerance in the drought-tolerant genotype BM14, important SUMs (carbohydrates, organic acids and phenylpropanoids) were mapped in metabolic pathway network according to literature and metabolic pathway databases (**Figure 8**).

## 4 Discussion

### 4.1 Osmoregulation and energy metabolism

Global spring wheat production is increasingly challenged by drought stress (Ceccarelli et al., 2010; Dong et al., 2018; Fan et al., 2018; Khan, 2019), highlighting the importance of elucidating physiological and metabolic mechanisms underlying drought tolerance. In the present study, we found that under osmotic stress, the drought-tolerant pure line BM14 maintained better growth, higher leaf water content, lower rate of water loss, and higher photosynthetic activity. Osmoregulation and energy metabolism play critical roles in plant drought tolerance (Gupta et al., 2020; Haghpanah et al., 2024; Xu and Fu, 2022). Under drought or high osmotic stress, plants typically accumulate inorganic ions and compatible solutes to lower intracellular water potential, thereby maintaining cellular water balance and structural stability. Previous studies have demonstrated that energy cost of accumulating inorganic ions is much lower than *de novo* synthesis of organic osmolytes to lower intracellular water potential (Hou et al., 2021; Shabala and Shabala, 2011; Zeng et al., 2015). In response to osmotic stress, plants primarily reduce cellular water potential through the uptake and accumulation of inorganic ions such as K^+^, Na^+^, NO_3_^-^, and Cl^-^ (Hou et al., 2021). In our study, the K^+^ content in the leaves of both genotypes significantly decreased under osmotic stress; however, the reduction was markedly smaller in BM14 than in NC4, with its K^+^ content being 2.67-fold higher than that in NC4. We also observed that many potassium channel genes and potassium transporter genes were upregulated in roots of BM14 but not in those of NC4 (unpublished manuscript), indicating that the elevated K^+^ accumulation is specific metabolic response strategy of BM14 to osmotic stress. Enhanced K^+^ accumulation has been widely associated with drought tolerance across diverse plant species (Feng et al., 2020; Wang et al., 2023; Sun et al., 2024; Li et al., 2024), revealing the role of K^+^ metabolism in drought tolerance is conserved among plants. In addition to accumulated higher concentration of K^+^ under osmotic stress, BM14 also exhibited genotype-specific accumulation for multiple types of metabolites. We focused on SUMs, and a total of 42 SUMs were identified in the leaves, including 14 carbohydrates, 6 flavonoids, and 3 phenolic acids. In the roots, 77 SUMs were identified, including 15 carbohydrates, 13 phenolic acids. These findings suggest that the enhanced accumulation of carbohydrates and flavonoids in leaves and the enhanced accumulation of carbohydrates and phenolic acids in roots, is a distinctive metabolic feature of BM14, which contributes to strong tolerance of this wheat genotype to osmotic stress. Free carbohydrates are well-recognized as primary organic osmolytes under drought stress and simultaneously function as key substrates for energy metabolism (Ozturk et al., 2021; Amoah and Adu-Gyamfi, 2025; Boriboonkaset et al., 2013). In our study, BM14 showed marked accumulation of multiple hub carbohydrates under osmotic stress, including fructose, galactose, glucose, sorbose and inositol in leaves, as well as trehalose, sucrose, panose and maltose in roots. Notably, under osmotic stress, sucrose accumulation was unaffected in NC4, but it was markedly upregulated in the roots of BM14. Sucrose has been widely recognized as a major organic osmolyte in response to salinity, osmotic, and drought stresses (Amoah and Adu-Gyamfi, 2025; Mathan et al., 2021; Suprasanna et al., 2016). To further explore the regulation mechanism by which osmotic stress induces sucrose synthesis in BM14, we assessed the activities of the key enzymes involved in sucrose synthesis. Consistent with enhanced sucrose accumulation, the activities of key sucrose synthesis enzymes SPS and SuSy were significantly elevated in the roots of BM14 under the stress condition, whereas their activities were unaffected in NC4. This coordinated upregulation of SPS and SuSy activities likely facilitates rapid sucrose accumulation, thereby supporting both osmotic adjustment and energy supply. Additionally, several important glycolysis-related carbohydrates (glucose, fructose, galactose, and sucrose) and intermediates of TCA cycle (malate, α-ketoglutarate, citrate, and isocitrate) were significantly upregulated in BM14 but remained unchanged in NC4. Similar enhancements in central carbon metabolism have been reported in other drought-tolerant crop genotypes, suggesting that strengthened energy metabolism represents a conserved adaptive trait that sustains stress responses under water deficit conditions (Li et al., 2024; Yin et al., 2024). These findings highlight energy metabolism-related pathways as promising targets for genetic improvement of drought resistance.

### 4.2 ROS homeostasis

**Figure 9:**
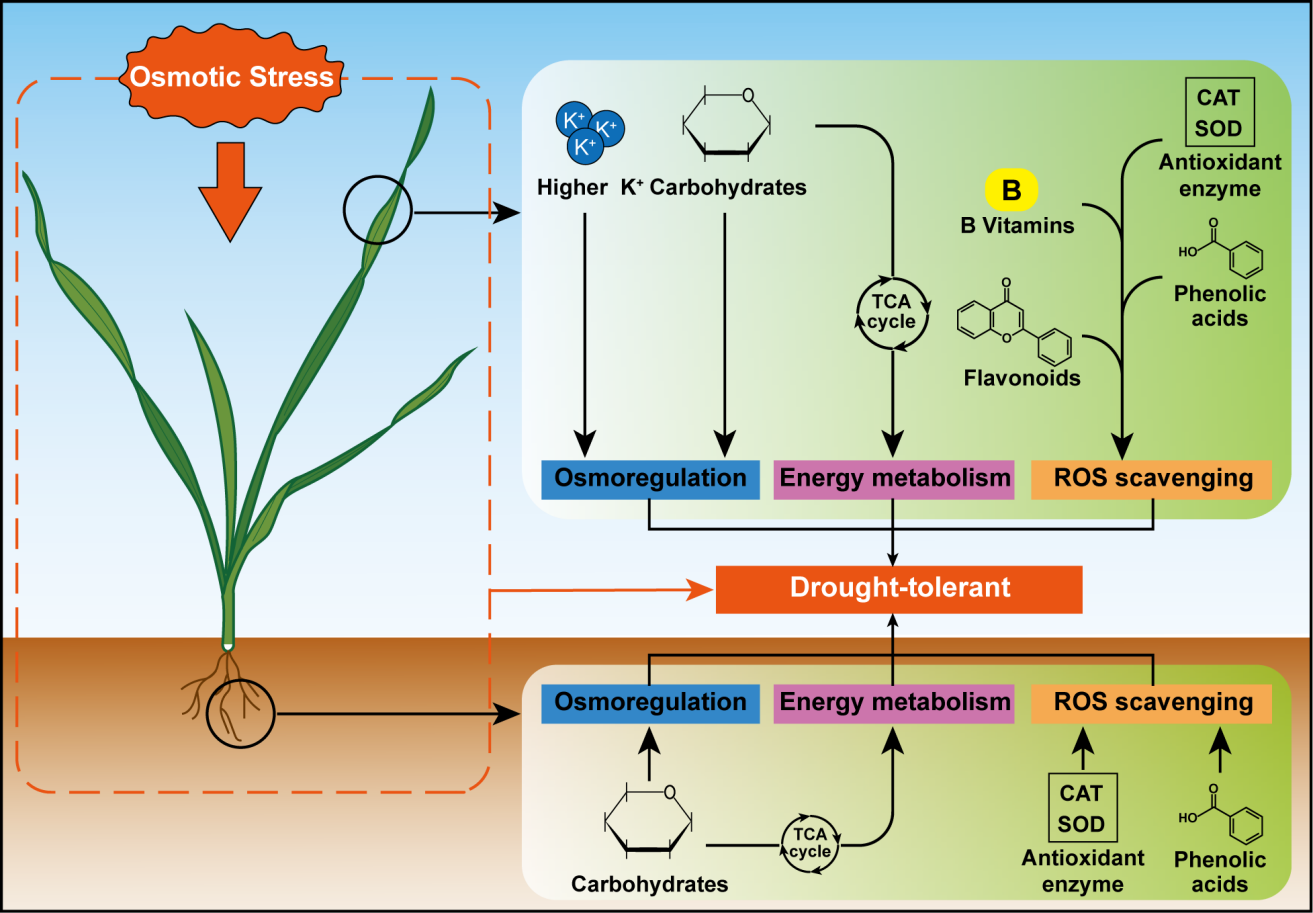
Organ-specific metabolic response strategies of drought-tolerant spring wheat genotype BM14 under osmotic stress.

Osmotic stress induces excessive accumulation of reactive oxygen species (ROS) in plants, leading to membrane lipid peroxidation, protein denaturation, and DNA damage, ultimately disrupting cellular structure and function (Bao et al., 2024; Hasanuzzaman et al., 2021). To mitigate ROS-induced damage, plants typically enhance antioxidant enzyme activities and accumulate non-enzymatic ROS scavengers (Hasanuzzaman et al., 2021; Irato and Santovito, 2021; Rao et al., 2025). In this study, we observed that under osmotic stress, the levels of O_2_^-^ in both leaves and roots of BM14 were significantly lower than those in NC4. Conversely, the activities of antioxidant enzymes, including SOD and CAT, were significantly higher in BM14 than in NC4 under osmotic stress. These findings indicated that enhanced antioxidant enzyme activity may contribute to the superior ROS-scavenging capacity of BM14, consistent with previous studies demonstrating the close association between antioxidant enzyme activity and drought tolerance in wheat (Abid et al., 2018; Ge et al., 2024; Hasanuzzaman et al., 2021). Regarding non-enzymatic antioxidants, BM14 exhibited pronounced organ-specific accumulation patterns in response to osmotic stress. These non-enzymatic antioxidants have been revealed to play critical roles in drought tolerance of other crops. For example, Wang et al. (2024b) reported that drought-tolerant soybean (*Glycine max* (L.) Merr.) varieties significantly increased the accumulation of flavonoids and phenolic acids to maintain ROS homeostasis under drought stress conditions. The inhibitive effect of abiotic stress on plant growth and metabolism has been associated with the degradation of B vitamins (Hanson et al., 2016). Although vitamins and plant hormones were discovered around the same time, vitamins have received considerably less attention than plant hormones in studies of abiotic stress (Hanson et al., 2016; Kohler, 1975). However, increasing evidence has revealed their vital antioxidant roles in plant stress responses (Asensi-Fabado and Munne-Bosch, 2010; Cao et al., 2024; Fitzpatrick, 2024). For instance, application of thiamine and pyridoxine has been shown to enhance antioxidant capacity and salt stress tolerance in faba bean (Ahmed and Sattar, 2024), and application of vitamin B2 (riboflavin) can improve drought tolerance in Agaricus bisporus (Guhr et al., 2017). Additionally, it had been reported that abiotic stress can upregulate B vitamin levels in tobacco plants (Huang et al., 2013), Arabidopsis thaliana (Rapala-Kozik et al., 2012), and maize (Rapala-Kozik et al., 2008). Based on these observations, we propose that B vitamins, together with phenolic acids and flavonoids, may mediate ROS scavenging and contribute to strong osmotic stress tolerance of BM14. Overall, our findings suggest that drought-tolerant spring wheat genotype employs organ-specific ROS scavenging strategies (**Figure 9**), relying on coordinated regulation of enzymatic and non-enzymatic antioxidants to maintain ROS homeostasis under osmotic stress.

Previous studies have reported that linoleic acid, α-tocotrienol, and L-leucine are closely associated with tolerance to PEG-induced osmotic stress in winter wheat (Li et al., 2022), while allantoin, galactaric acid, gluconic acid, and glucose have been linked to drought tolerance in rice (Ghorbanzadeh et al., 2023). In line with these findings, our results demonstrate that the drought-tolerant spring wheat genotype responds to osmotic stress through enhanced accumulation of key glycolysis-related carbohydrates, tricarboxylic acid (TCA) cycle intermediates, B vitamins, phenolic acids, and flavonoids, indicating a distinct metabolic response strategy in spring wheat. Although PEG-6000 treatment does not fully reproduce the complexity of soil drought, particularly with respect to root–soil and root–microorganism interactions, it effectively induces osmotic stress, a major component of soil drought. Consequently, PEG-based treatments may bias the identification of differentially accumulated metabolites (DAMs), especially in roots, compared with soil-based drought conditions. Nevertheless, both our results and soil drought–based studies consistently highlighted the importance of K^+^ homeostasis, carbohydrate metabolism, and phenolic acids and flavonoids in osmotic stress or drought tolerance, supporting robust reliability of the metabolic traits based on PEG treatment in the present study (Chen et al., 2025; Wang et al., 2025; Yan et al., 2025; Cui et al., 2019; Xu et al., 2021; de Souza Mateus et al., 2022).

## 5 Conclusion

Different organs of the drought-tolerant spring wheat genotype BM14 exhibited different strategies responsive to osmotic stress (**Figure 9**). For osmoregulation and energy metabolism, BM14 accumulated higher concentrations of K^+^ and carbohydrates in the leaves, while it accumulated higher concentrations of carbohydrates in the roots. During response to osmotic stress, phenolic acids were major non-enzymatic antioxidants in the roots of BM14, while flavonoids, phenolic acids, and B vitamins were major non-enzymatic antioxidants in the leaves. These findings suggest that BM14 alleviates negative effects of osmotic stress through coordinated increases in leaf K^+^ levels, enhanced energy metabolism and organ-specific ROS scavenging strategies.

## Data Availability

The original contributions presented in the study are included in the article/**Supplementary Material**. Further inquiries can be directed to the corresponding author.

## Supplementary material

Supplementary Table 1 Complete statistical information for all detected metabolites and SUMs obtained from pairwise group comparisons, including metabolite annotation, fold change, log_2_ fold change, variable importance in projection (VIP), raw P-values, and Benjamini–Hochberg (BH)–adjusted p-values. BCL, BM14 leaves under control conditions; BSL, BM14 leaves under osmotic stress; NCL, NC4 leaves under control conditions; NSL, NC4 leaves under osmotic stress; BCR, BM14 roots under control conditions; BSR, BM14 roots under osmotic stress; NCR, NC4 roots under control conditions; NSR, NC4 roots under osmotic stress.
