# Supplementary material for: Metabolic response strategies of spring wheat under osmotic stress
Source: Front Plant Sci. 2026 Feb 10;17:1766233. doi: 10.3389/fpls.2026.1766233 (PMC12929553; doi:10.3389/fpls.2026.1766233)
Supplement: Supplementary Table 1 — Complete statistical information for all detected metabolites and SUMs obtained from pairwise group comparisons, including metabolite annotation, fold change, log2 fold change, variable importance in projection (VIP), raw P-values, and Benjamini–Hochberg (BH)–adjusted p-values. BCL, BM14 leaves under control conditions; BSL, BM14 leaves under osmotic stress; NCL, NC4 leaves under control conditions; NSL, NC4 leaves under osmotic stress; BCR, BM14 roots under control conditions; BSR, BM14 roots under osmotic stress; NCR, NC4 roots under control conditions; NSR, NC4 roots under osmotic stress. [file Supplementaryfile1.docx]

Supplementary Material


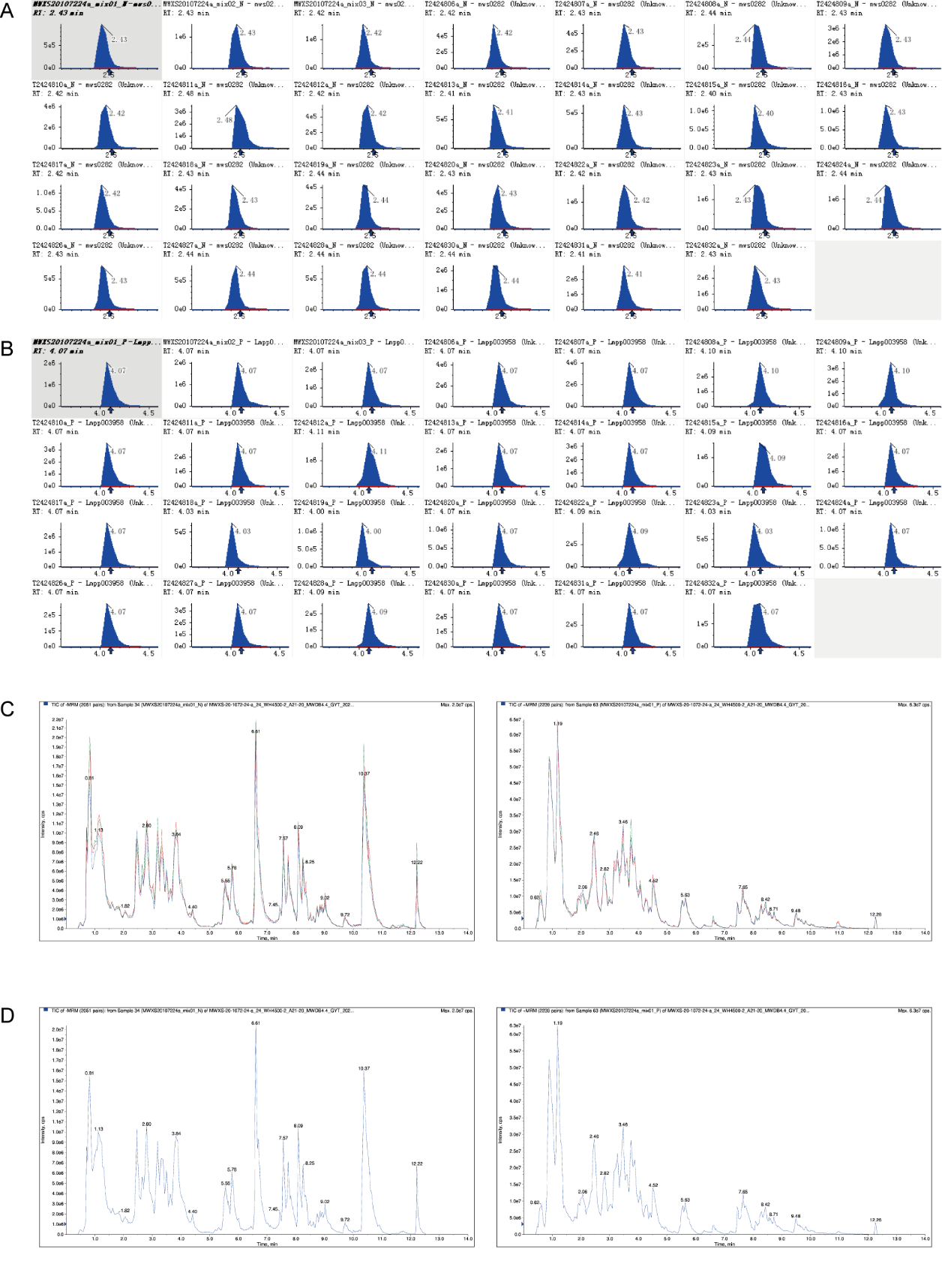


**Supplementary Figure 1.** Metabolite quantification and quality control analysis. **(A, B)** Extracted ion chromatograms (EICs) showing peak area integration for metabolite quantification. The abscissa represents the retention time (min) of metabolite detection, while the ordinate represents the ion current intensity (cps) of a specific metabolite ion detection. The peak area indicates the relative content of the substance in the sample. **(C)** Overlay of TICs from QC samples, demonstrating the stability and reproducibility of the mass spectrometry system. **(D)** Total ion chromatogram (TIC) of a quality control (QC) sample obtained by UPLC-MS/MS analysis.


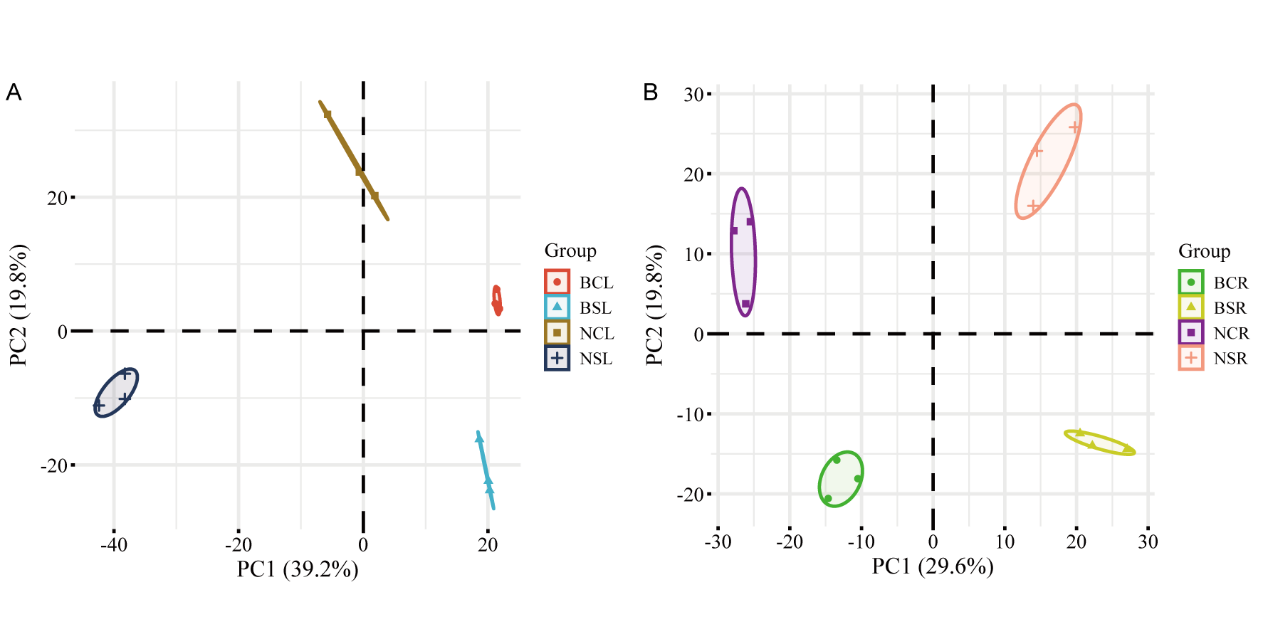


**Supplementary Figure 2.** Principal Component Analysis of metabolic profiles of two spring wheat genotypes under control and osmotic stress. **(A)** Leaf PCA; **(B)** Root PCA. BCL, BM14 leaves under control; BSL, BM14 leaves under osmotic stress; NCL, NC4 leaves under control; NSL, NC4 leaves under osmotic stress; BCR, BM14 roots under control; BSR, BM14 roots under osmotic stress; NCR, NC4 roots under control; NSR, NC4 roots under osmotic stress.


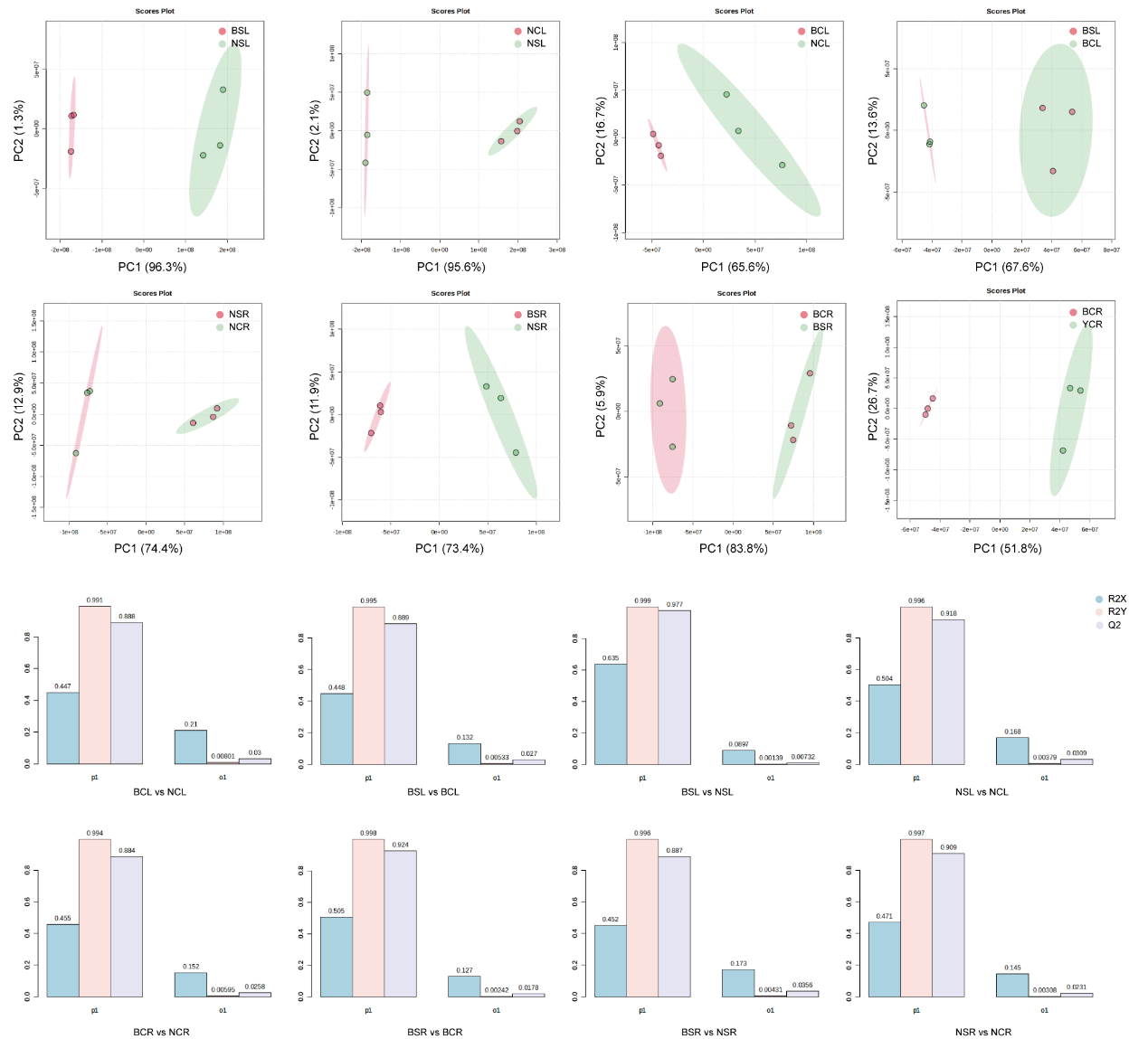


**Supplementary Figure 3.** OPLS-DA score plots and model diagnostics of metabolomics data. **(A)** OPLS-DA score plot, showing the distribution of samples between groups. **(B)** Model diagnostics of the OPLS-DA analysis, including R²X, R²Y, and Q² values, used to evaluate model quality and predictability. BCL, BM14 leaves under control; BSL, BM14 leaves under osmotic stress; NCL, NC4 leaves under control; NSL, NC4 leaves under osmotic stress; BCR, BM14 roots under control; BSR, BM14 roots under osmotic stress; NCR, NC4 roots under control; NSR, NC4 roots under osmotic stress.


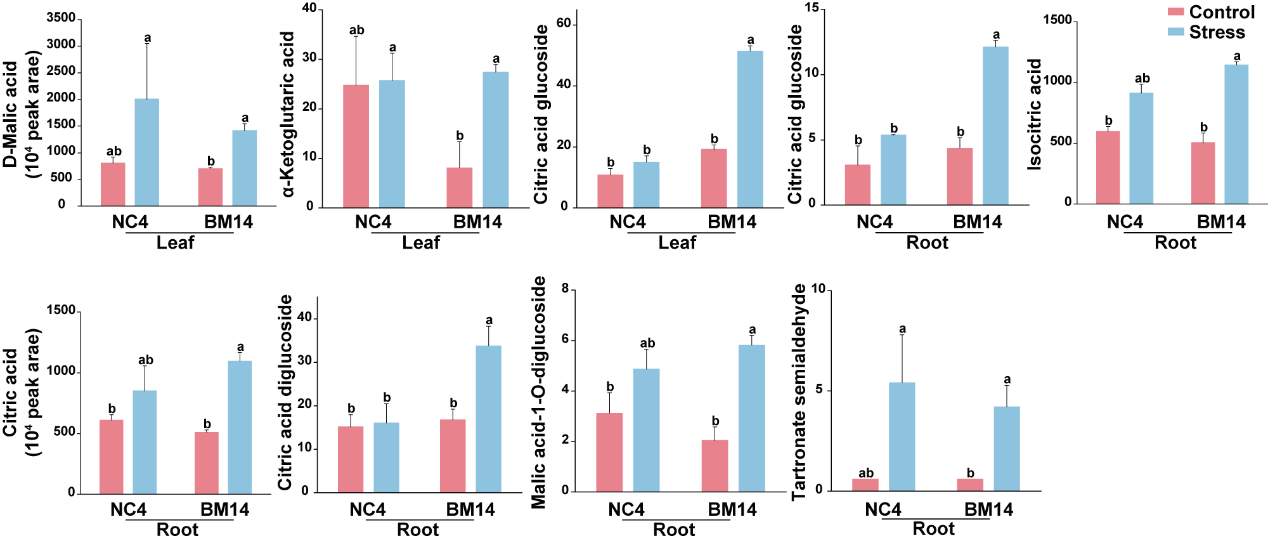


**Supplementary Figure 4.** Effects of osmotic stress on relative concentration of organic acids in leaves and roots. Data are presented as means ± SD of three biological replicates (n = 3). Different letters indicate significant differences among treatments (VIP > 1, |log₂(FC)| > 1, and FDR < 0.05).


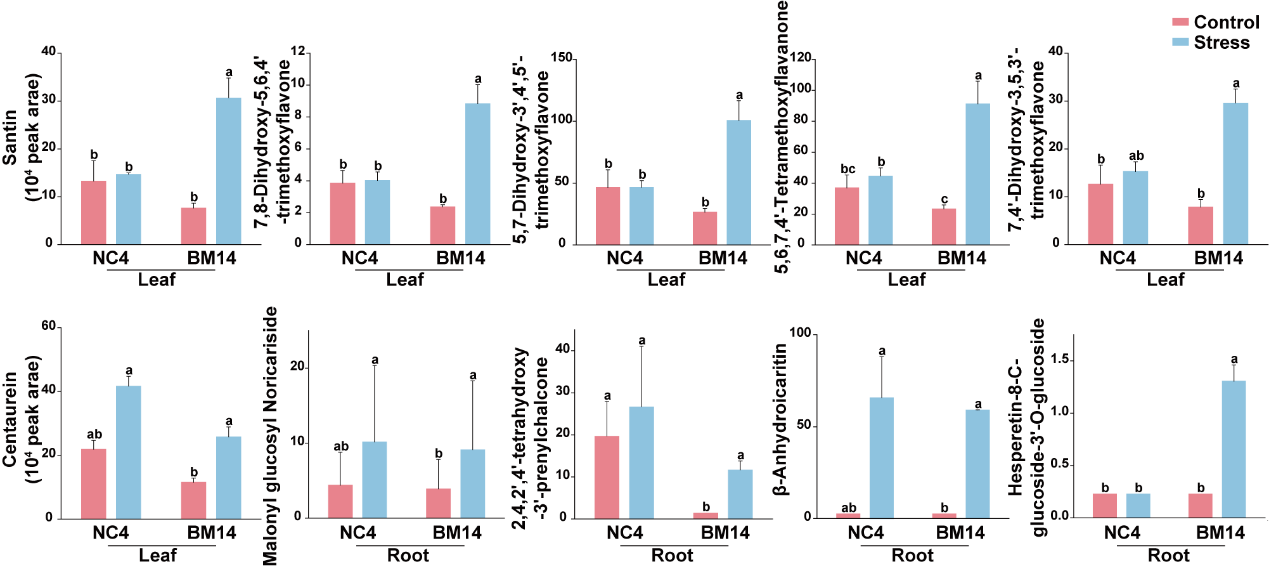


**Supplementary Figure 5.** Effects of osmotic stress on relative concentration of flavonoids in leaves and roots. Data are presented as means ± SD of three biological replicates (n = 3). Different letters indicate significant differences among treatments (VIP > 1, |log₂(FC)| > 1, and FDR < 0.05).


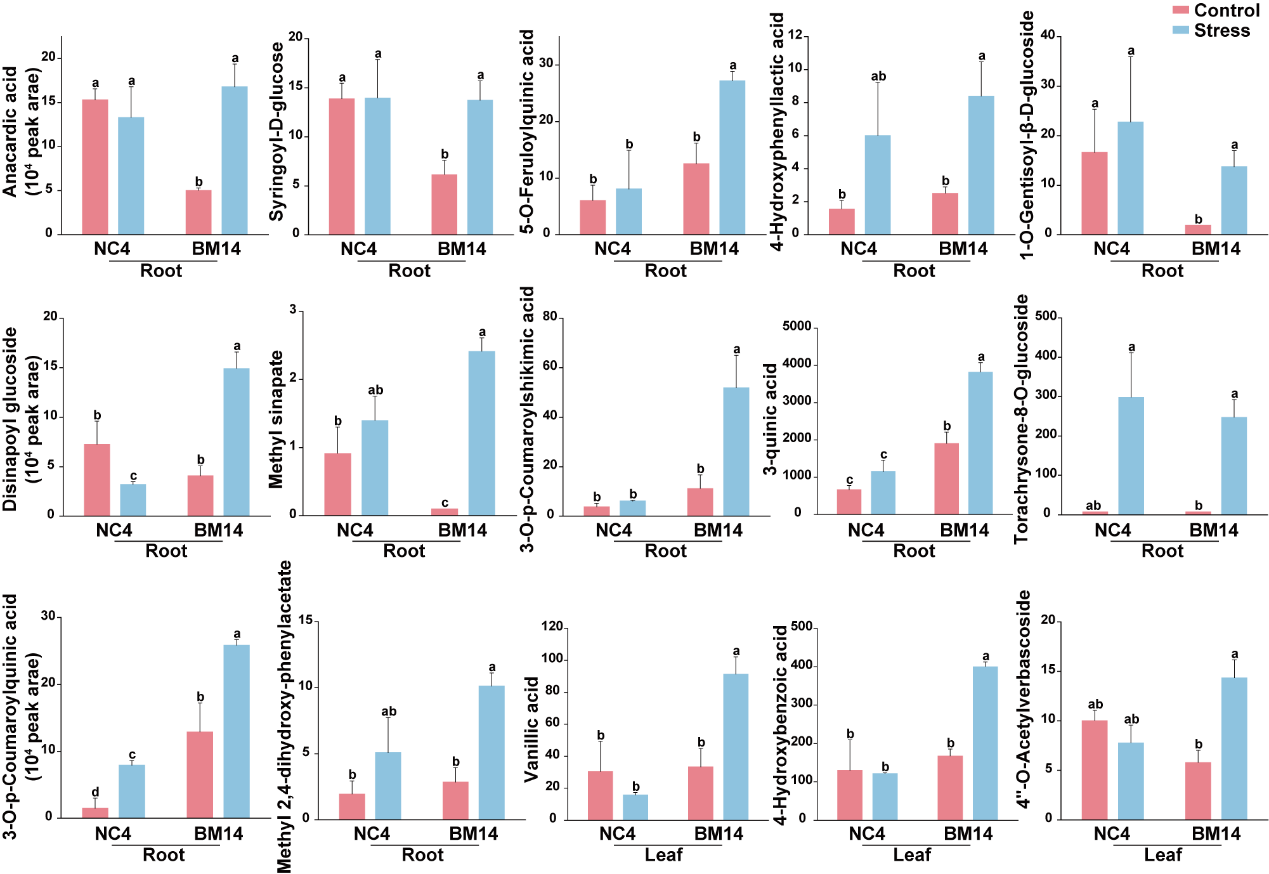


**Supplementary Figure 6.** Effects of osmotic stress on relative concentration of phenolic acids in leaves and roots. Data are presented as means ± SD of three biological replicates (n = 3). Different letters indicate significant differences among treatments (VIP > 1, |log₂(FC)| > 1, and FDR < 0.05).


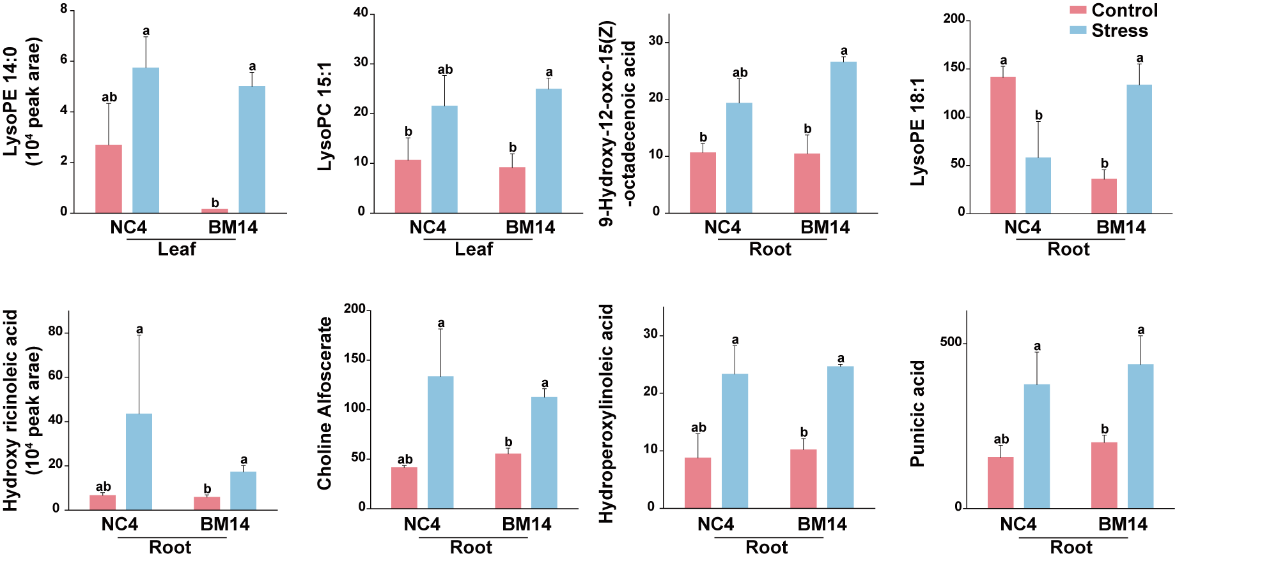


**Supplementary Figure 7.** Effects of osmotic stress on relative concentration of lipids in leaves and roots. Data are presented as means ± SD of three biological replicates (n = 3). Different letters indicate significant differences among treatments (VIP > 1, |log₂(FC)| > 1, and FDR < 0.05).


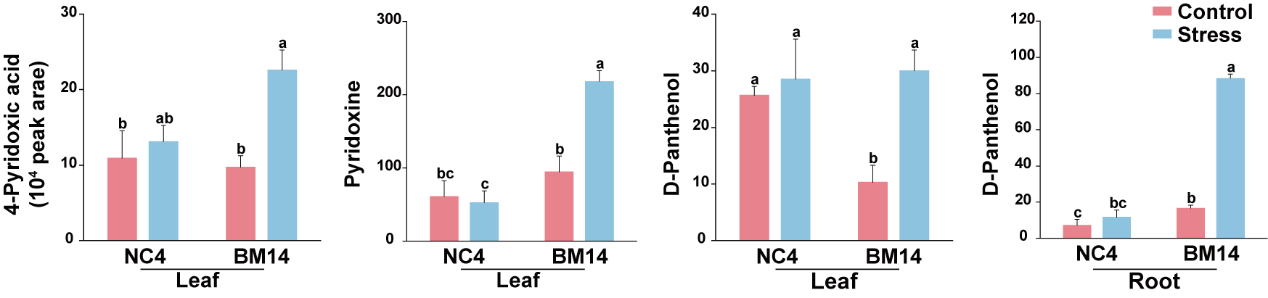


**Supplementary Figure 8.** Effects of osmotic stress on relative concentration of Vitamin in leaves and roots. Data are presented as means ± SD of three biological replicates (n = 3). Different letters indicate significant differences among treatments (VIP > 1, |log₂(FC)| > 1, and FDR < 0.05).
